# Academic Medical Missions – Reaching Those in Greatest Need?

**DOI:** 10.5334/aogh.2651

**Published:** 2019-10-15

**Authors:** Keerteshwrya Mishra, Sonal Jessel, Jacob Lurie, Kane O. Pryor, Gunisha Kaur

**Affiliations:** 1 Wayne State University School of Medicine, US; 2 Columbia University, US; 3 Icahn School of Medicine at Mount Sinai, US; 4 Weill Cornell Medicine, US

To the Editor,

Academic institutions in developed countries are increasingly participating in medical missions abroad [1]. Medical students and resident physicians across all specialties have expressed growing interest in international clinical rotations as a means to understand the global burden and epidemiology of diseases, global healthcare disparities, and biopsychosocial influences on health [2]. Medical anthropologists have hypothesized however that in addition to the potential unintended consequences of medical missions, academic institutions do not actually participate where need is the greatest but rather in more well-developed regions. We investigated whether or not such a gap exists between academic medical mission location and countries in greatest need.

Poverty, health, and human development indicator data were used as a measurement of healthcare need and were gathered from the World Bank, United Nations, and World Health Organization [3]. Specifically, maternal mortality ratios, life expectancy at birth, and the composite human development index, established indicators of health and wellbeing, were mined from 2015–2017 (the last year for which data were available was used for each category). The top 25 countries ranking worst globally in each category were delineated. From these data, a composite list of the top most disadvantaged countries was generated, resulting in 38 unique countries in need.

**Figure 1:**
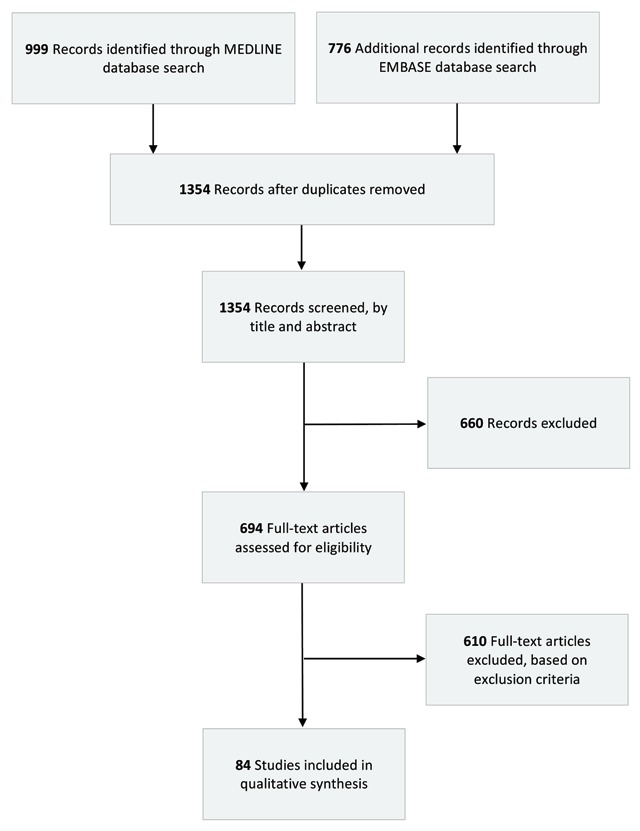
PRISMA Style Flow Diagram of Systematic Review.

Simultaneously, MEDLINE and EMBASE were queried from 2013 to 2017 to identify articles published by academic physicians participating in medical missions, as a surrogate indicator of where missions occur. MeSH terms included medical missions, relief work, volunteer, student, medical, short-term medical mission, abroad, and international. 1,354 un-duplicated results were screened for title and abstract yielding 694 articles for full text review. Of these, 84 articles underwent complete analysis (Figure 1). Key data such as country visited, country of origin, type of service, funding, and clinical specialty of physicians, were extracted and characterized based on country visited.

**Figure 2:**
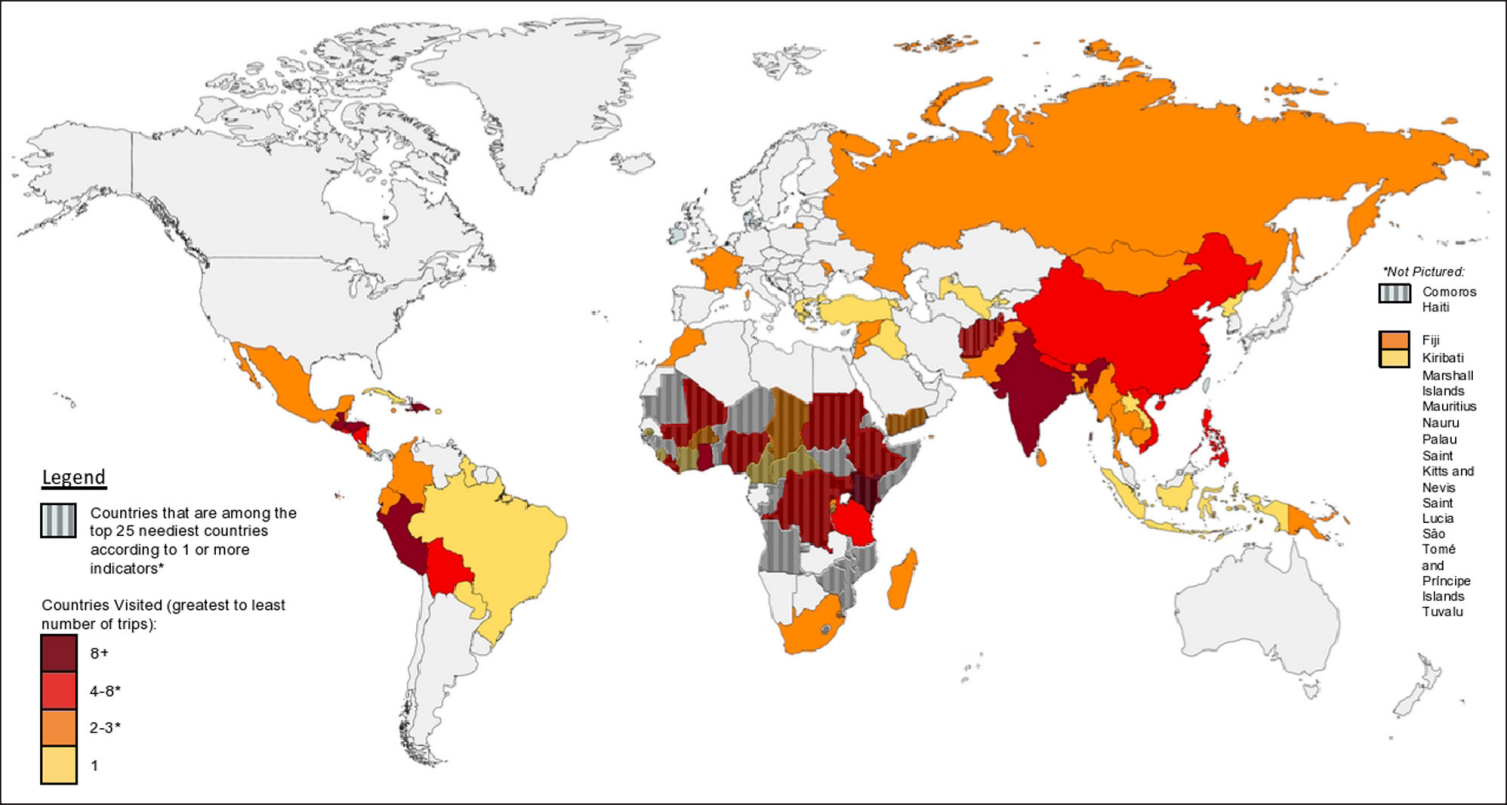
Countries visited as compared to countries in greatest need by poverty, health, or human development indicator data.

The incidence of academic medical missions in each country globally as determined by systematic review of MEDLINE and EMBASE was mapped onto the composite list of countries most in need by poverty, health, and human development indicators. Additionally, medical mission occurrence was overlaid on income category for each country as defined by the World Bank (Figure 2).

107 academic medical missions were reported over the five-year period reviewed. Of these interventions, only 29% (n = 31) occurred in a country ranking in greatest need. An additional 6% (n = 6) of interventions took place in these countries during emergency situations such as the 2015 Ebola pandemic in West Africa. 32% (n = 34) of all academic missions occurred in low-income countries, 51% (n = 55) in lower-middle income countries, 15% (n = 16) in upper-middle income countries, and 2% (n = 2) in high-income countries.

The involvement of trainees in global health electives, with appropriate education, may result in improved medical knowledge and diagnostic skills, increased awareness of the social determinants of health, enhanced cultural understanding, exposure to a wide spectrum of illnesses, and appreciation of resource utilization [4]. Yet without adequate education, medical work abroad may challenge local providers and healthcare infrastructure, deliver context insensitive teaching, drain precious local resources, and compromise patient care when trainees are unsupervised [5]. As such, the conduct of international electives, particularly those that involve trainees, should occur when the benefits of participation are balanced with the risks of intervention.

Our data indicate that potentially only a fraction of academic medical missions occur in the world’s most disadvantaged countries, where need and benefit of intervention might be greatest. Reasons for this might include the safety profile of a region, longstanding partnerships with local institutions, and ease of travel, and should be investigated further. The investigation was limited by the use of publications in academic journals as a surrogate for interventions by academic medical missions.

Where academic medical missions occur is significant, because it directs the academic and training focus of the medical community, particularly in developed countries where investment in global education and teaching is the greatest. Since the Declaration of Alma Ata in 1978 that established healthcare as a human right, the focus of global health leaders has been on increasing access to care. Recently, the community has been reflecting on quality assurance, care of non-communicable diseases, and populations that have been impacted by extreme violence, mass migration, and natural disasters. The data presented here on the practice of medical care in resource poor settings by academic physicians and trainees are a critical component of the narrative.
